# Use of contrast enhanced ultrasound in testicular diseases: A comprehensive review

**DOI:** 10.1111/andr.13057

**Published:** 2021-06-11

**Authors:** Marta Tenuta, Franz Sesti, Ilaria Bonaventura, Paola Mazzotta, Riccardo Pofi, Daniele Gianfrilli, Carlotta Pozza

**Affiliations:** ^1^ Department of Experimental Medicine Sapienza University Rome Italy

**Keywords:** acute scrotum, CEUS, infertility, testicular tumor, testis

## Abstract

**Background:**

Contrast‐enhanced ultrasound (CEUS) is a sonographic technique that increases the diagnostic accuracy of ultrasound and color Doppler ultrasound (CDUS) when studying testicular abnormalities. However, its role in clinical practice is still debatable because there are no accepted standards regarding how and when this technique should be used for patients with testicular disease.

**Objectives:**

To perform a nonsystematic review of the current literature to highlight the strength and flaws of performing CEUS and to provide a critical overview of current research evidence on this topic.

**Materials and methods:**

A thorough search of published peer‐reviewed studies in PubMed was performed using proper keywords.

**Results:**

Strong enhancement of neoplastic lesions (both benign and malignant) during CEUS aids in differential diagnosis with non‐neoplastic lesions, which usually appears either nonenhanced or enhanced in a manner similar to that of the surrounding parenchyma. CEUS enhancement has a high predictive value in the identification of neoplastic lesions, whereas a similar or complete absence of enhancement may be interpreted as strong evidence of benignity, although there are exceptions. Literature on quantitative analysis is still scarce, though promising, particularly in distinguishing benign from malignant neoplasms. Furthermore, CEUS may be useful in many emergency situations, such as acute scrotum, blunt scrotal trauma, and focal infarction of the testis. Finally, CEUS can help increase the probability of sperm recovery in azoospermic males.

**Discussion and conclusion:**

CEUS is a safe, easy‐to‐perform, and cost‐effective diagnostic tool that can provide a more accurate diagnosis in testicular lesions and acute scrotal disease. However, further studies with larger cohorts are required to refine the differential diagnosis between benign and malignant neoplasms. Finally, these preliminary results can instigate the development of innovative research on pre‐testicular sperm extraction to increase the chances of sperm recovery.

## 1 INTRODUCTION

Testicular ultrasound (US) currently represents a routine and mandatory investigation for patients with scrotal symptoms and is considered the first‐line imaging modality in the evaluation of the testis and adjacent structures, in addition to physical examination. Since its introduction, US has become an integral diagnostic tool in clinical settings that has been further enhanced by continuous developments to improve the resolution of US machines and probes.^1^, ^2^, ^3^

Although grayscale US, color Doppler US (CDUS), and power Doppler US (PDUS) demonstrate high diagnostic accuracy for detecting most testicular pathologies, interpretation of the acquired images is not well standardized and may rely on the operators’ expertise. Recently, the use of certain techniques such as tissue elastography and magnetic resonance imaging (MRI)^4^, ^5^, ^6^, ^7^, ^8^, ^9^, ^10^ has been explored in terms of overcoming this limitation, as well as superb microvascular imaging (SMI), which specifically aims to visualize low velocity and small diameter blood vessel flow.^11^, ^12^, ^13^ However, their strength is still debatable.

In the last few decades, the use of intravascular contrast‐enhanced US (CEUS) has grown considerably and has proved to be a useful tool in many diagnostic fields.^14^, ^15^, ^16^, ^17^, ^18^

US contrast medium consists of US‐detectable microbubbles, which are very small‐sized (<10 μm) organic shells that are filled with gas. Over the years, many US contrast agents have been approved for clinical use,^17^, ^19^ such as sulfur hexafluoride (SonoVue^®^, Bracco, Milan, Italy), octafluoropropane, (Definity^®^, Lantheus Medical Imaging, North Billerica, MA, USA), perfluorobutane (Sonazoid™, GE Healthcare, Oslo, Norway), and perflutren protein‐type A microsphere (Optison™, GE Health care, Oslo, Norway).

A dedicated machine‐setting with a low mechanical index (0.05–0.08) is needed to avoid early microbubble destruction.^20^ The localization of microbubbles is exclusively intravascular because they are small enough to pass through the lumina of capillaries, yet large enough to prevent extravasation from vessels. Due to their high impedance, they reflect the majority of US waves with a higher echo than the parenchyma. In fact, unlike CDUS and PDUS, CEUS provides a reliable representation of blood perfusion and parenchymal microcirculation in various organs, using intravascular blood tracers. After injection of the contrast medium, two phases are described in organs with a single arterial blood supply^20^: the first is the arterial phase (10–40 s), which shows a progressive enhancement; the second one is the venous phase (30–45 s), which starts after injection and exhibits a plateau followed by a progressive decrease, until the microbubble signal completely disappears.

CEUS offers a number of advantages: it is easy to perform, cost‐effective, safe, and does not have any harmful effects compared to other complementary imaging methods such as CT and MRI. First, the number of allergic reactions reported is lower than those arising with CT and MRI contrast medium.^21^, ^22^, ^23^ The overall reporting rate for all adverse events is 0.125% (only 0.0086% for serious ones)^23^ including itching, mild dizziness, moderate hypotension, headache, and nausea which resolved spontaneously. Second, CEUS is neither cardiotoxic nor nephrotoxic and can be safely administered in patients with renal insufficiency because the contrast medium is not excreted via the kidney, but it is cleared by the lungs.^20^, ^24^ Consequently, and due to all the other advantages offered, this diagnostic tool can also be used on children.^25^, ^26^ Finally, compared to MRI, CEUS offers higher spatial resolution (especially using new high frequency probes, up to 18 MHz), allowing for a dynamic assessment even of smaller lesions.

Over the last years, CEUS has proved to be particularly useful in testicular setting: microbubbles trace normal parenchymal microcirculation and are able to highlight intraparenchymal abnormalities within the testicle. This is particularly useful in the characterization of testicular lesions^7^, ^8^, ^9^, ^10^, ^27^, ^28^, ^29^, ^30^, ^31^, ^32^, ^33^ and acute scrotum.^34^, ^35^, ^36^ Recent studies have also focused on the utility of CEUS in evaluating testicular perfusions prior to testicular sperm extraction (TESE) in infertile men.^37^, ^38^ However, to date, there are no well‐established and accepted standards with respect to how and when this technique should be used when dealing with patients suffering from testicular disease.

The purpose of this study was to perform a comprehensive, up‐to‐date review of the current literature to highlight the strength and flaws of performing CEUS, and to provide a critical overview of current research evidence on this topic to inform and guide clinicians’ choices of performing CEUS in certain conditions, and to provide them support in the interpretation of the exam.

A computerized literature search was performed using the following keywords: “CEUS,” “testicle,” “testicular tumor,” “testicular lesion”, “seminoma”, “Leydig cell tumor”, “scrotal trauma,” “testicular torsion”, and “infertility”. Keywords were properly combined with Boolean operators to optimize the search strategy.

## 2 TESTICULAR CEUS TECHNIQUE

CEUS can depict parenchymal disorders on the basis of vascularity, thus mostly helping in the differential diagnosis of traumatic changes and scrotal lesions. For testicular studies, the most frequently used contrast agent in Europe is sulfur hexafluoride (SonoVue^®^). SonoVue is injected as two intravenous boluses of 2.4 mL (for a total of 4.8 mL) in an antecubital vein. The second dose should be injected 5–10 min after the first injection, and both should be followed immediately by 10 mL of 0.9% saline solution. After contrast medium and saline solution flush, microbubbles are usually observed within the testicle after a mean time of 20 s,^20^ thus enabling clinicians to draw an exact vascular map of the examined testis. The enhancement of testicular and epididymal arteries occurs rapidly, followed by a subsequent parenchymal enhancement.

**FIGURE 1:**
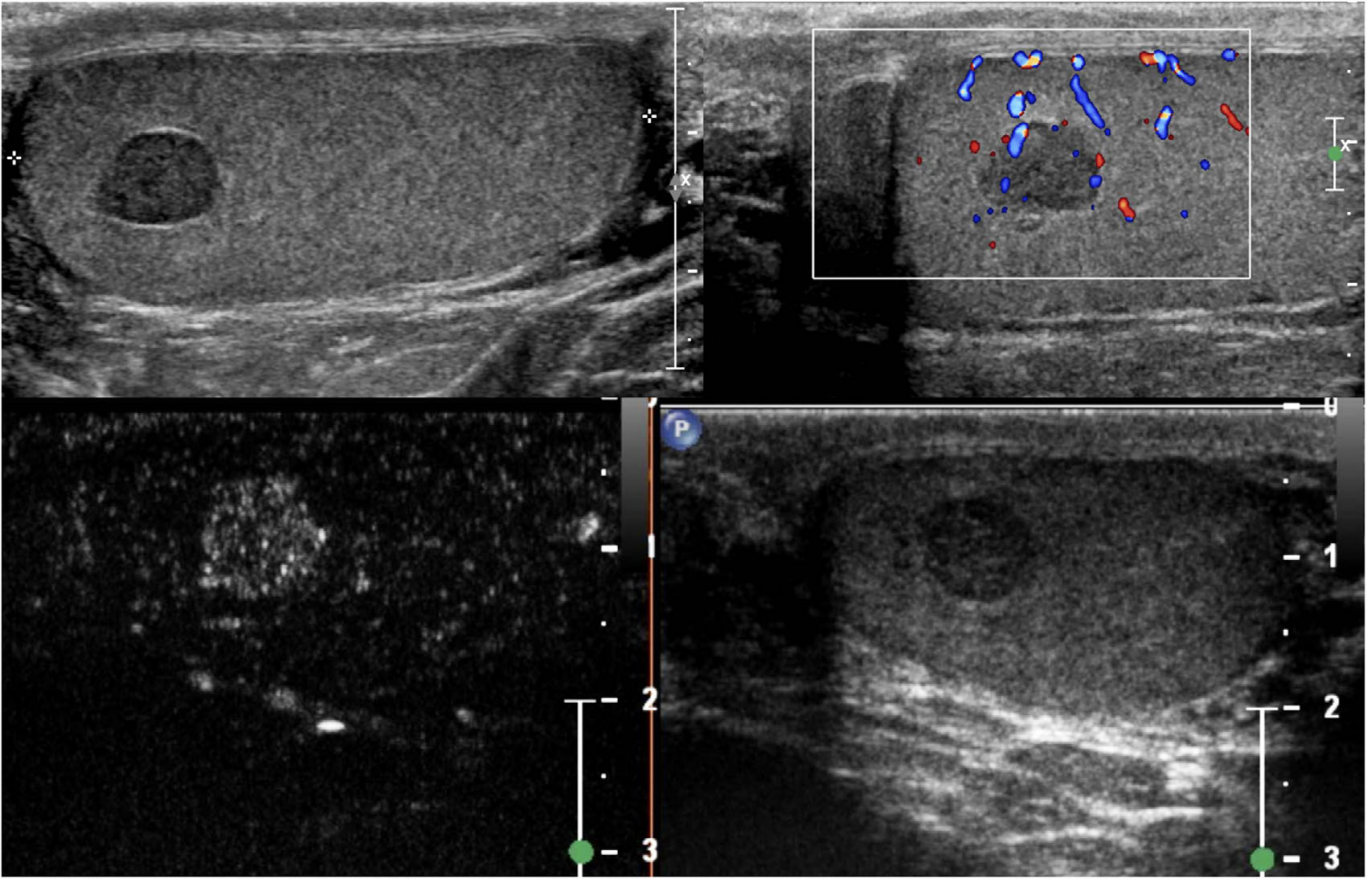
Qualitative analysis of CEUS. B‐mode US demonstrates a small hypoechoic lesion, with hyperechoic and well‐defined margins, resulted a Leydig cell tumor at histology. Color Doppler US demonstrates vascularity within the lesion. With contrast‐enhanced US, the lesion demonstrates marked hyperenhancement, a characteristic that has the potential to differentiate neoplastic from nonneoplastic lesions

Typically, the contrast medium is no longer visible after an average of 3–5 min.^20^ The entire examination needs to be recorded for subsequent analyses. Recording should be initiated at the end of each contrast‐enhancement injection and should be concluded after at least 90 s. The first evaluation that can be performed involves a qualitative analysis: after each injection, it is possible to observe whether the contrast medium enhances the area of interest, and subsequently evaluate the intensity and timing of the uptake (wash‐in), and release (washout) of microbubbles compared with parenchyma. The area of interest can be defined as hyper‐enhancing (Figure 1), hypo‐enhancing, or non‐enhancing compared with the surrounding parenchyma, and the wash‐in and washout can be defined as faster, similar, or slower than parenchyma.^7^, ^9^, ^27^, ^28^, ^29^, ^31^ However, qualitative analysis is subjective and operator‐dependent. In contrast, quantitative analysis using appropriate software generally integrated in the US machine is a significantly less biased approach.

**FIGURE 2:**
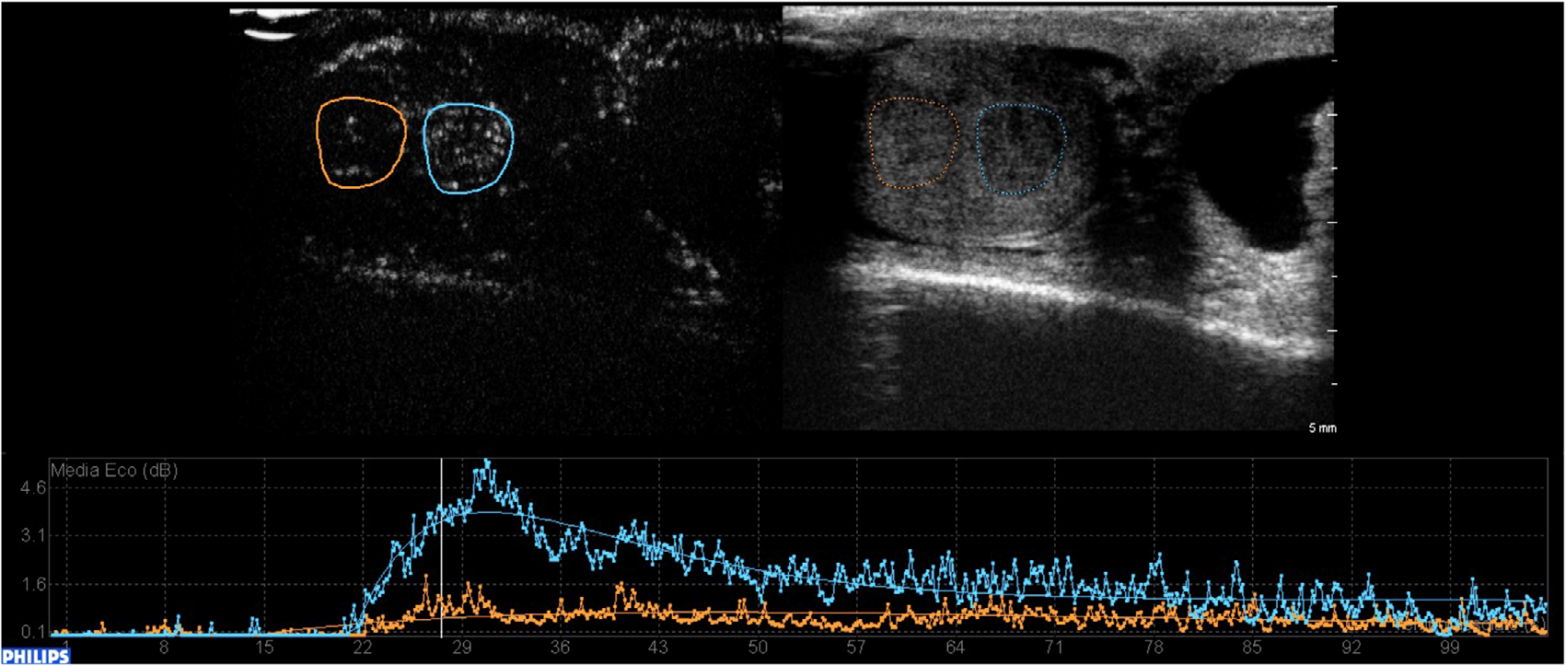
Time intensity curves (TIC). They are bell‐shaped curves that describe an initial uptake phase of the contrast medium (wash‐in) up to the maximum peak of intensity, and a subsequent release phase (washout). Lesion and parenchyma kinetics can be measured, and the resulting curves can be compared. In the figure the blue curve describes the wash‐in and wash‐out phases of a Leydig cell tumor, the orange curve describes the phases of the adjacent, normal parenchyma

Time‐intensity curves can be obtained by manually placing a region of interest (ROI) to entirely cover the area to be examined. Another identical ROI should be placed on the adjacent parenchyma for comparison.^28^ Within the ROI, the mean intensity of contrast enhancement can be described as a function of time with time‐intensity curves: they are bell‐shaped curves that describe an initial uptake phase of the contrast medium (wash‐in) up to the maximum peak of intensity, and a subsequent release phase (washout) (Figure 2).

**FIGURE 3:**
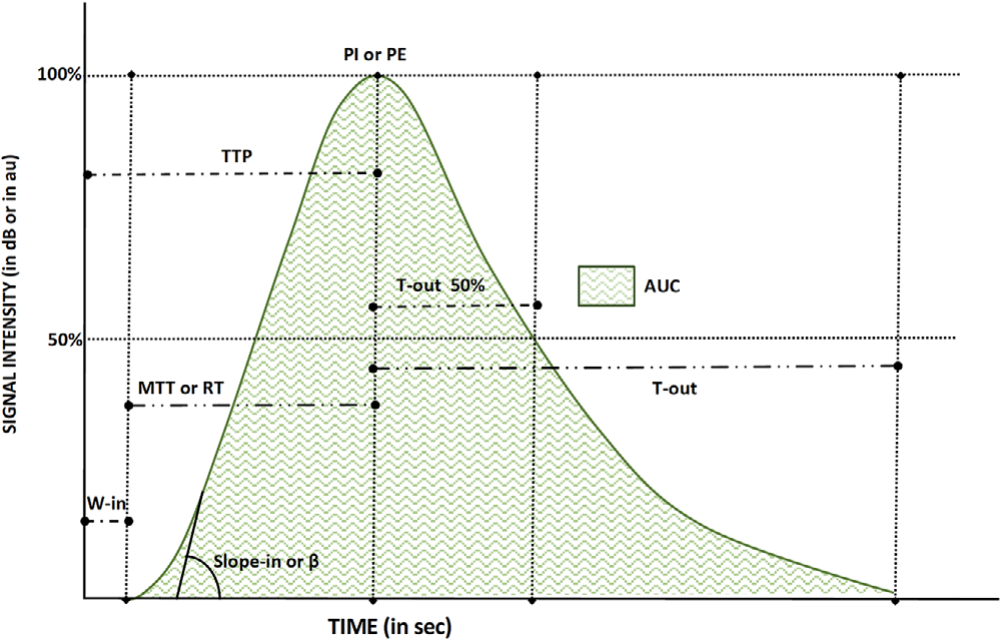
Graphic representation of the time/intensity curve and the calculated perfusion parameters—*wash‐in time (W‐in)*: the time when testis enhancement first occurs, measured in seconds; *time to peak (TTP)*: the time needed to reach the *peak intensity (PI)*, measured in seconds; *mean transit time (MTT) or rise time (RT)*: the time difference between the time needed to reach the PI and the time since the beginning of ROI enhancement, measured in seconds; PI *or peak enhancement (PE)*: the maximum ROI enhancement, measured in decibel (dB) or acoustic units (au); *washout time (T‐out)*: the time difference between the 50% PI values in the washout and peak intensity value, measured in seconds (several studies also consider T‐out as the time needed for the descending slope to reach a contrast signal intensity of zero); *area under the curve (AUC)*: intensities of the entire enhancement period, measured in dB or au. Several studies also differentiate wash‐in AUC (before PI) and washout AUC (after PI); *slope in or β*: the coefficient of the wash‐in slope, it reflects the mean blood flow velocity in the region of interest, measured in dB or au

Wash‐in and washout of the contrast agent can be quantified by calculating intensity and temporal parameters (quantitative parameters). Normal testicular parenchyma time‐intensity curve values are lacking in literature, and existing studies focus on focal lesion kinetics. However, depending on the software used, the parameters obtained may be different, and different units of measurement could also be employed, particularly for intensity data. In general, it is possible to identify certain standard values, which are essential for the subsequent quantitative analysis^7^, ^28^, ^29^, ^31^, ^38^, ^39^ (Figure 3):

1. Wash‐in time (W‐in): the time when testicular enhancement occurs first, measured in seconds
2. Time to peak (TTP): the time needed to reach the peak intensity, measured in seconds
3. Mean transit time (MTT) or rise time (RT): the difference between the time needed to reach the peak intensity and the time since the beginning of ROI enhancement, measured in seconds
4. Peak intensity (PI) or peak enhancement (PE): the maximum ROI enhancement, measured in decibel (dB) or acoustic units (au)
5. Washout time (T‐out): the time difference between the 50% PI values in the washout and PI value, measured in seconds. Several studies also consider T‐out as the time needed for the descending slope to reach a contrast signal intensity of zero
6. Area under the curve (AUC): intensities throughout the entire time of enhancement, measured in dB or au. Several studies also differentiate wash‐in AUC (before PI) and washout AUC (after PI)
7. Slope in or β: the coefficient of the wash‐in slope, which reflects the mean blood flow velocity in the ROI, measured in dB or au

## 3 CEUS AND TESTICULAR LESIONS

One of the most successful uses of CEUS reported in the literature involves the differential diagnosis of intratesticular lesions.^33^ Males presenting a palpable testis nodule are likely to have malignant germ‐cell tumor in > 90% of cases. However, the increased use of testicular US as a diagnostic tool in most andrological pathologies and the recent developments of high‐frequency probes in ultrasonography have allowed for an increase in the detection of small, incidental intratesticular lesions that are thought to be benign in > 30% of cases. Thus, a radical orchiectomy should be considered as overtreatment.^40^

The first step for clinicians is to distinguish whether a lesion is neoplastic or non‐neoplastic. If a neoplastic lesion is suspected, the second step is to differentiate between benign and malignant testicular tumors (TTs). Clinical history (genetic syndromes, history of cryptorchidism, previous surgery, infertility, previous contralateral tumor, and familiarity for testicular cancer), symptoms (sudden or chronic pain and swelling), and laboratory data (serum tumoral markers) can significantly assist this process. However, in certain cases, the differential diagnosis can still be challenging. During the last decades, CEUS has become a very useful method to improve the characterization of nonpalpable testicular lesions, and its use is recommended by the European Federation of Societies for Ultrasound in Medicine and Biology (EFSUMB) guidelines.^20^, ^41^

### 3.1 CEUS in the differential diagnosis between non‐neoplastic and neoplastic intratesticular lesions

Non‐neoplastic intratesticular lesions include simple cyst, epidermoid cyst, segmental ischemia, abscess, hematomas, post biopsy scars, orchitis, adrenal rest tumors, and sarcoidosis. Their US and CDUS characteristics are reported in Table 1. The use of CDUS alone can be adequate to perform a differential diagnosis, because the majority of non‐neoplastic testicular lesions are nonvascular, with the exception of focal orchitis, sarcoidosis, and adrenal rest tumors (TART).^42^, ^43^ However, particularly in the case of small lesions, CDUS alone can often fail to depict internal vascularization. Blood flow, indeed, cannot be detected if the transducer is not positioned at a right angle to the vessels, or in case of very small vessels with low volume blood flow. CEUS can overcome these limitations, thus providing a reliable representation of lesion's microcirculation. This is because US contrast medium can reach even smaller vessels,^10^ therefore confirming the presence of vascularity or the nonenhancement of a lesion.^44^

**TABLE 1:** Ultrasound, CDUS and CEUS characteristic of principal non‐neoplastic intratesticular lesions

	Non‐neoplastic intratesticular lesions			
	Grayscale ultrasound	CDUS	CEUS	CEUS literature
Simple cyst	Rounded anechoic lesions with hyperechoic rim	Avascular	Unenhanced	Auer et al., 2011^10^ Isidori et al., 2014^24^
Epidermoid cyst	Well‐circumscribed rounded lesion with “onion ring” aspect (with concentric rings of hypoechogenicity and hyperechogenicity) or densely calcified mass or cyst with peripheral rim/central calcification or mixed atypical pattern	Avascular	Unenhanced/perilesional rim enhancement	Auer et al., 2011^10^ Lock et al., 2011^23^ Patel et al., 2012^41^ Isidori et al., 2014^24^ Schroder et al., 2016^8^ Anheuser et al., 2019^42^ Schwarze et al., 2020^7^ Lung et al., 2020^83^
Segmental infarction	Hypoechoic area with undefined margins, generally with a lobular shape	Avascular	Unenhanced/perilesional rim enhancement	Auer et al., 2011^10^ Parenti et al., 2012^73^ Isidori et al., 2014^24^ Patel et al., 2014^71^ Lorenz et al., 2019^75^ Lung et al., 2020^83^
Abscess	Complex heterogeneous fluid collection with irregular walls, low level internal echoes	Avascular/vascular rim	Unenhanced/perilesional rim enhancement	Isidori et al., 2014^24^ Schroder et al., 2016^8^ Lung et al., 2020^83^
Post biopsy scar	Oval or triangular hypoechoic area beneath the albuginea	Avascular	Unenhanced	Auer et al., 2011^10^ Schroder et al., 2016^8^
Hematoma	Well‐circumscribed hypoechoic lesions with areas of high reflectivity. Size decrease in time is typical	Avascular	Unenhanced/perilesional rim enhancement (rarely)	Lobianco et al., 2011^31^ Hedayati et al., 2012^68^ Yusuf et al., 2015^72^ Lung et al., 2020^83^
Focal orchitis	Single or multiple hypoechoic areas	Vascularized	Hyperenhanced	Auer et al., 2011^10^ Isidori et al., 2014^24^ Lung et al., 2020^83^
Adrenal rest	Hypoechoic lesions with irregular margins, hyperechogenic foci, typically localized in the mediastinum testis (generally bilateral)	Vascularized	Hyperenhanced	Corcioni et al., 2021^52^
Sarcoidosis	Hypoechoic lesions with irregular margins (often bilateral)	Vascularized	Hypoenhanced	Lung et al., 2020^83^

CDUS: color Doppler ultrasound; CEUS: contrast‐enhanced ultrasound.

**FIGURE 4:**
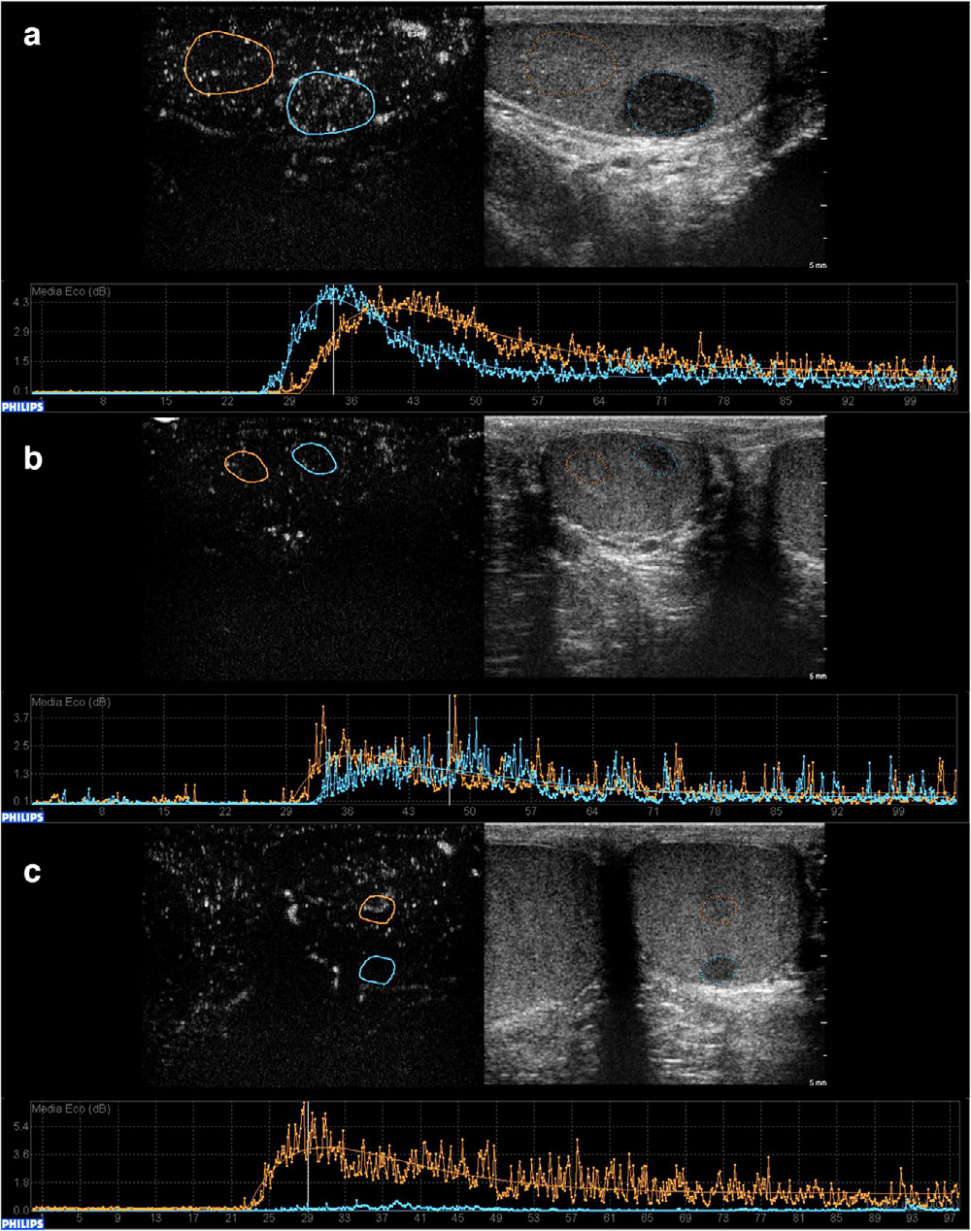
CEUS in the differential diagnosis between non‐neoplastic and neoplastic intratesticular lesions. (a) A hyperenhanced lesion compared to the adjacent parenchyma, turned out to be a seminoma at histology. (b) A hysoenhanced lesion compared to the parenchyma resulted a Leydig cell hyperplasia at histology. (c) A hypoenhanced lesion, resulted focal fibrosis at definitive histology

According to several published studies, neoplastic lesions (both benign and malignant) tend to be strongly enhanced during CEUS, thus facilitating the differential diagnosis with non‐neoplastic lesions, which, in general, are either nonenhanced or enhanced in a similar manner to that of the surrounding parenchyma (Figure 4).

The first study in this field dates back to 2011.^27^ The authors prospectively described the feasibility of CEUS in the differential diagnosis of testicular masses. Findings revealed that in 50 out of the 51 patients examined, focal lesions demonstrated a different contrast enhancement compared to the surrounding testicular tissue. In detail, testicular lesions in 39 (76.5%) patients revealed hyperenhancement, of which 38 (97.4%) were diagnosed histologically as neoplasms. It is noteworthy that early arterial hyperenhancement was predictive of a neoplastic lesion, with a sensitivity of 88.4% (95% CI: 74.1–95.6%) and a positive predictive value of 97.4% (95% CI: 84.9–99.9%).^27^ Hyperenhancement was not found in 7/8 lesions, which proved to be non‐neoplastic. These results were confirmed by a subsequent prospective study on 67 patients in which hyperenhancement in CEUS showed a sensitivity of 93% and a predictive positive value of 96% for detecting testicular neoplasms.^8^ Subsequent retrospective studies confirmed the high sensitivity, specificity, and positive predictive value of CEUS in identifying neoplastic lesions,^10^, ^32^ which were significantly higher than those of CDUS alone. More specifically, according to Auer et al., CDUS showed a sensitivity of 66.7%, a specificity of 88.4%, a correct classification rate of 83.6%, and a positive likelihood ratio of 5.7 (*p* < 0.001).^10^ In case of testicular epidermoid cysts, CEUS validated the complete absence of contrast bubbles within the lesion and consequently the pathognomonic absence of internal vascularization.^45^, ^46^

A description of the uptake kinetics was performed for the first time by Isidori et al. on 115 patients consisting of 38% patients with malignant tumors, 37% with benign tumors, and 25% with non‐neoplastic lesions. Non‐neoplastic lesions revealed a wash‐in that was similar or more delayed to the parenchyma compared to all tumors (76% vs. 35%, *p* < 0.001) as well as a similar washout (76% vs 21%, *p *< 0.001).^28^

**FIGURE 5:**
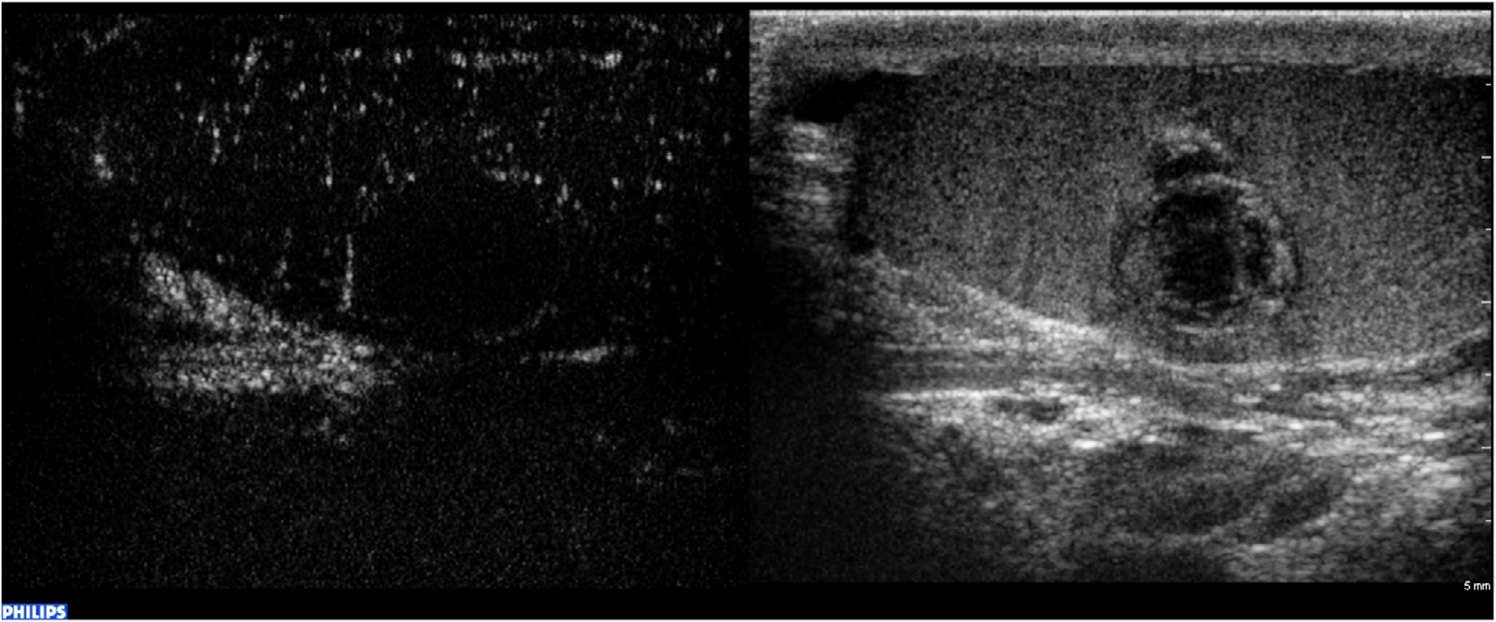
Epidermoid cyst. B‐mode US demonstrates a well‐circumscribed, solid, mixed‐reflectivity lesion with high‐reflectivity “onion‐skin” peripheral rims. Contrast‐enhanced US demonstrates a clear lack of enhancement within the lesion

To sum up, lesions that were more enhanced compared to the surrounding parenchyma seem to have a higher predictive value in identifying neoplastic lesions, whereas similar enhancement or its complete absence can be interpreted as strong evidence for benignity.^10^, ^42^ However, there are some exceptions, represented by epidermoid cysts (Figure 5), necrotic embryonal carcinoma, and burned out tumors (BOT), all neoplastic lesions that are typically not vascularized internally. In the latter conditions, B‐mode imaging, clinical history, and examination are essential for reaching a correct diagnosis. According to CEUS kinetics, wash‐in and washout, similar or delayed to the parenchyma, can be suggestive of non‐neoplastic lesions.

### 3.2 CEUS in the differential diagnosis between benign and malignant neoplastic lesions

TTs are rare neoplasms that account for approximately 1–1.5% of all human cancers. However, TTs represent the most common neoplasm in males between 15 and 44 years who are in full reproductive age.^47^ TT can be primarily distinguished into germ‐cell tumors and non‐germ‐cell tumors.^48^ Germ‐cell tumors are almost always malignant, whereas non‐germ‐cell tumors are most commonly stromal tumors with a benign behavior. Recent evidence has shown that the frequency of stromal tumors is probably underestimated, particularly when they are small. In fact, according to recent series, the incidence could be significantly higher (3–22%)^28^, ^31^, ^40^, ^49^, ^50^, ^51^ than the one reported in previous research studies. In certain cases, malignant tumors can also undergo regression, necrosis, and scarring while spreading with distant metastasis, as forBOT.^51^ Finally, although rarer, tumors of lymphatic or hematopoietic origin with intratesticular localization should also be described.^30^, ^52^

Conventional US demonstrates high sensitivity for TT detection, yet this diagnostic method offers low specificity in differentiating benign from malignant lesions.^53^ The appearance of TTs in US may change according to histology. A classification of TTs^48^ and their most commonly associated B‐mode and CDUS features is provided in Table 2. In most cases, stromal cell tumors are Leydig cell tumors (LCT), which usually appear as a small, unique, hypoechoic, homogeneous, and well‐demarcated lesion. Sertoli cell tumors are much less common and can appear as both hypo‐ and hyper‐echoic lesions, with possible intralesional calcifications. Among malignant testicular neoplasms, seminomatous tumors usually appear as focal lesions, hypoechoic to the normal surrounding parenchyma, with irregular margins, whereas nonseminomatous tumors are usually heterogeneous with internal calcification or cystic areas. However, large seminomatous tumors can also appear inhomogeneous and can involve the whole parenchyma of the testicle. Moreover, US features such as intralesional calcifications, irregular/infiltrating margins, and the presence of parenchymal microlithiasis are usually associated with malignancy.^7^, ^28^

**TABLE 2:** Ultrasound, CDUS, and CEUS characteristic of principal neoplastic intratesticular lesions

	Neoplastic intratesticular lesions				
	Grayscale ultrasound	CDUS	CEUS	CEUS Literature	
Leydig cell tumor	Hypoechoic, homogeneous well‐demarcated lesion	Hypervascularized	Homogeneously hyperenhanced	Auer et al., 2011^10^ Lock et al., 2011^23^ Lock et al., 2014^9^ Cantisani et al., 2012^62^ Isidori et al., 2014^24^ Drudi et al., 2015, 2016^5^	Schroder et al., 2016^8^ Luzurier et al., 2019^27^ Lerchbaumer et al., 2019 Pozza et al., 2019^36^ Lung et al., 2020^83^ Schwarze et al., 2020^7^
Sertoli cell tumor	Both hypo‐ and hyper‐echoic lesions, with possible calcifications	Hypervascularized	Homogeneously hyperenhanced	Auer et al., 2011^10^ Isidori et al., 2014^24^ Luzurier et al., 2019^27^	Lerchbaumer et al., 2019 Lung et al., 2020^83^ Schwarze et al., 2020^7^
Seminoma	Hypoechoic round or oval lesion, occasionally multinodular or with polycyclic lobulated margins	Hypervascularized	Homogeneously hyperenhanced	Auer et al., 2011^10^ Lock et al., 2011^23^ Isidori et al., 2014^24^ Luzurier et al., 2019^27^ Drudi et al., 2015, 2016^25^	Schroder et al., 2016^8^ Peil Grum et al., 2018^48^ Lerchbaumer et al., 2019 Schwarze et al., 2020^7^ Lung et al., 2020^83^
Embryonal cell carcinoma	Hypoechoic heterogeneous lesions which can present internal cystic areas or calcific margins and distal acoustic shadowing	Hypervascularized/ avascular	Hyper‐hypo‐unenhanced	Isidori et al., 2014^24^ Lerchbaumer et al., 2019	Lung et al., 2020^83^ Schwarze et al., 2020^7^
Teratoma	Heterogeneous lesions, well‐circumscribed, predominantly cystic with hyperechoic spots	Hypervascularized	Inhomogeneously hyperenhanced	Isidori et al., 2014^24^	Lung et al., 2020^83^
Choriocarcinoma Yolk sak tumor	Heterogeneous lesions with hypo‐anechoic areas (hemorrhage, necrosis) and calcifications	Hypervascularized	Hyperenhanced	Schwarze et al., 2020^7^	
Mixed germ‐cell tumor	Different aspect in regard to main histological component	Hypervascularized	Homogeneously/inhomogeneouslyhyperenhanced	Lock et al., 2011^23^ Isidori et al., 2014^24^	Lung et al., 2020^83^ Schwarze et al., 2020^7^
Burned out tumor	Highly echogenic foci or gross calcifications/ hypoechoic irregular areas	Hypovascularized	Unenhanced	Lock et al., 2011^23^ Isidori et al., 2014^24^	Rocher et al., 2016^47^ Luzurier et al., 2019^27^
Lymphoma	Hypoechoic lesions with diffuse infiltration or multifocal hypoechoic lesions of various size	Hypervascularized	Hyperenhanced	Lock et al., 2011^23^ Isidori et al., 2014^24^ Lock et al., 2016 Schroder et al., 2016^8^	Peil Grum et al., 2018^48^ Schwarze et al., 2020^7^ Lung et al., 2020^83^
Leukemia	Diffuse or focal, hypoechoic or hyperechoic with infiltrating pattern	Hypervascularized	Hyperenhanced	Schwarze et al., 2020^7^	

CDUS: color Doppler ultrasound; CEUS: contrast‐enhanced ultrasound.

According to previous reports, increased vascularization (with arborization and branches) has been considered to be a malignant tumor characteristic.^54^, ^55^ Nonetheless, vascularization is not specific for malignant diagnosis because it can also be increased in stromal tumors,^40^ focal orchitis,^10^ TARTs,^56^ or benign mesenchymal tumors such as capillary hemangioma^57^, ^58^, ^59^ and leiomyoma.^60^ Particularly, LCTs can appear as having a more intense blood flow than seminomas.^9^, ^29^

For this reason, distinguishing a malignant tumor from a benign neoplasm between incidental lesions is a significantly challenging task, particularly for small, hypoechoic, and well‐vascularized masses with regular margins. In particular, LCTs and seminomas can be very similar on nonenhanced US.^61^, ^62^ Performing an accurate and careful differential diagnosis is imperative because both benign and malignant tumors have a very different clinical course. Patients suspected of benign lesions can be addressed to tissue‐sparing surgery enucleation or, in selected cases, to clinical and US strict surveillance, thus preserving the testicle instead of performing total orchiectomy,^63^, ^64^ which is suggested in case of malignancies.

In this perspective, CEUS could represent as an additional and effective tool. To date, there have been only a few prospective studies that tried to evaluate whether the use of CEUS could help in the differential diagnosis between benign and malignant TTs.^7^, ^28^, ^29^, ^31^ Results are promising, however, the available data are not always in agreement, and the majority of reports are based on qualitative rather than a more objective quantitative assessment.

As reported earlier, hyperenhancement is the most common feature observed in TT on CEUS.^28^, ^29^, ^31^ The current literature underlines that benign lesions are characterized by lower enhancement; however, in these reports, both neoplastic and non‐neoplastic lesions are included in the benign group.^7^, ^10^, ^32^ It must be considered, though, that some malignant lesions have an architecture that does not allow the uptake of the contrast medium. In fact, this is the case in BOT, where malignant cells are rapidly replaced by fibrotic tissue.^28^, ^31^, ^51^ Luzurier et al. demonstrated how CEUS could help differentiate BOT from vascularized TTs.^31^ Similarly, some malignant tumors with large intralesional necrotic areas or embryonal carcinoma with calcific margins may demonstrate hypo‐enhancing features.^3^, ^28^, ^65^

As already pointed out, qualitative analysis can guide the clinician's evaluation. However, this process is based heavily upon the operator's experience; thus, the subjective interpretation of US images can be biased. In contrast, quantitative analysis can provide more objective data, yet research in this field is still substantially limited, and studies performed have used small sample sizes and have results that are not reproducible and/or not always comparable because of the use of different measurement units.^33^

Isidori et al. were the first to compare the kinetic parameters between malignant and benign lesions (both neoplastic and non‐neoplastic). According to the authors, rapid wash‐in and washout were distinctive characteristics of malignant lesions (77% vs. 25%, *p* < 0.001), whereas similar or delayed wash‐in (compared with the parenchyma) were appropriate signs of benign lesions (both neoplastic and non‐neoplastic) (61% vs. 20.5%, *p* < 0.001). Subsequent quantitative analysis revealed that TTP, MTT, and T‐out were all significantly shorter in malignant tumors than in benign ones, as wash‐out time appeared to be slower in benign lesions.

**FIGURE 6:**
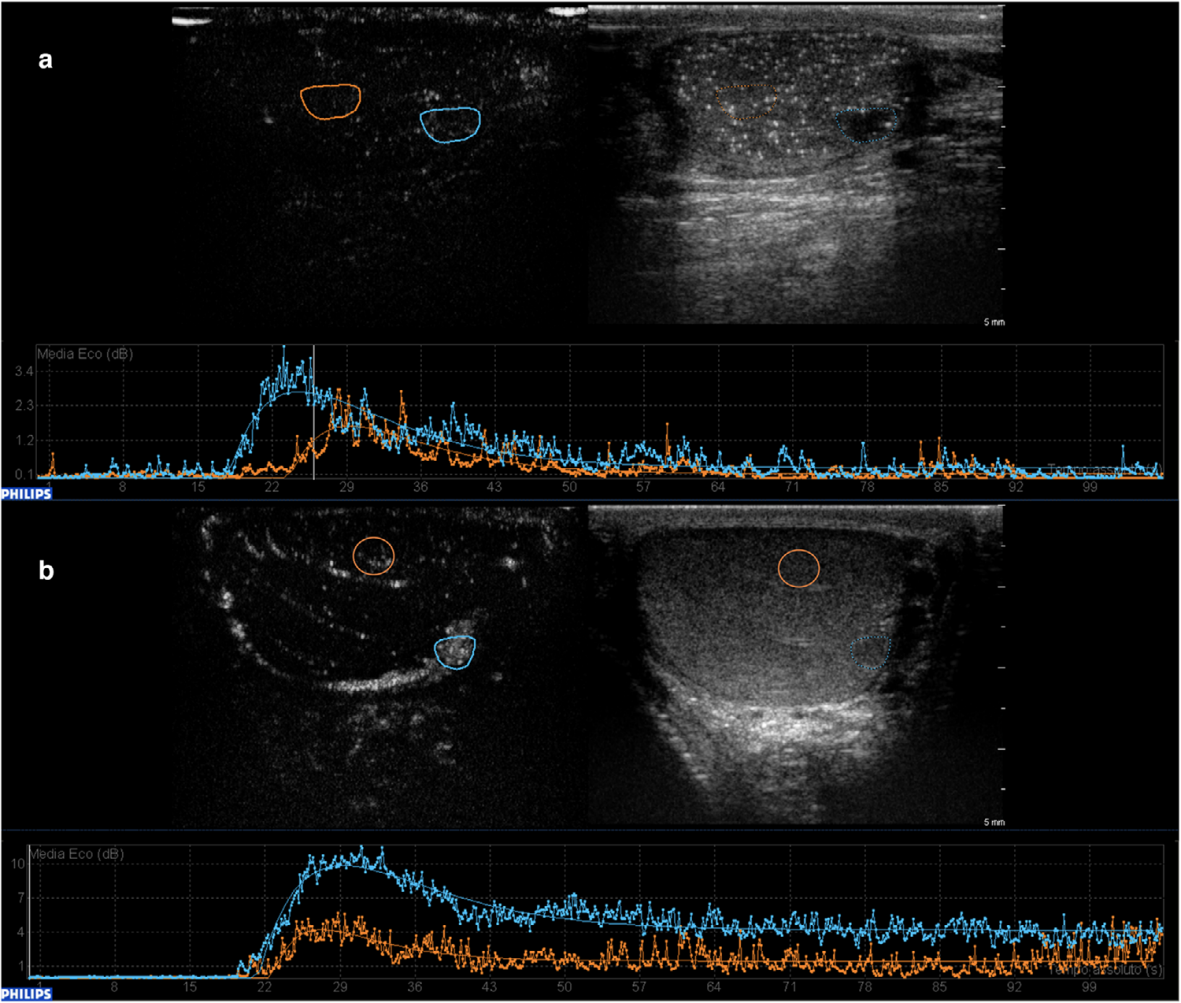
Quantitative analysis comparison of a seminoma and a Leydig cell tumor. A rapid wash‐in and wash‐out are distinctive characteristics of seminomas, as demonstrated by the blue curve in panel (a) that shows a wash‐in that begins approximately at 18 s and a wash‐out starting at 30–35 s, whereas a rapid wash‐in (20 s) and a delayed wash‐out (starting at 38–40 s) are appropriate signs of a Leydig cell tumor (panel b, blue curve). The orange curves belong to the adjacent normal parenchyma

The authors also performed a comparison between the two largest histologically proved homogeneous groups (malignant seminomas and stromal tumors) obtaining similar results^28^ (Figure 6). In this prospective study, CEUS application supported the authors in selecting the appropriate patient intervention, since 25 of 115 patients underwent US strict surveillance with serial investigations every 3 months for a minimum of 18 months, instead of surgery, without any disease progression at the final follow‐up. The same was described in the study by Pozza et al., where 32 of 83 patients did not undergo surgery because CEUS, ES, MRI and clinical findings were suggestive for LCTs, therefore US strict surveillance was performed. All patients were disease‐free at the final visit, thus suggesting that in compliant patients, active surveillance through clinical and radiological follow‐up could be a safe alternative option for small nonpalpable lesions suspicious for LCTs at CEUS.^40^

More recently, Drudi et al. performed a comparative analysis on seminoma and LCT.^29^ Interestingly, LCT showed a faster TTP and greater PI and WiR. Similar findings have been previously reported in a series of 13 LCTs^9^ and in one case report.^66^ The authors attributed these results to the vascular architecture of LCT, which is characterized by a wider and more regular vascular bed and a greater microvessel density compared to seminomas. In fact, according to Samson et al., LCT has a vessel density that is 3.2‐fold higher than seminoma. This could be explained by the expression of endocrine gland‐derived vascular endothelial growth factor (EG‐VEGF), which is strongly expressed in Leydig cells, and specifically in LCT, as opposed to germ‐cell tumors which do not express this angiogenic factor.^67^

According to Schwarze et al., PI was greater in malignant tumors, whereas wash‐in was found to be faster in benign lesions. However, it should be emphasized that the authors of this study included both neoplastic and non‐neoplastic among benign lesions.^7^ Finally, according to Luzurier et al., CEUS failed in providing an effective differential diagnosis between benign and malignant tumors, as no differences were found in any parameter during the quantitative analysis. However, when excluding BOTs (considered a separate group), the sample size of this work remained significantly small (out of 31 lesions, 15 were malignant and 13 were benign).^31^

The results are hardly comparable due to the heterogeneity of the examined lesions (even within the same subgroup) and to the software used to obtain kinetic parameters, which produced results that were expressed in different and noncomparable scales.^33^

## 4 CEUS BEYOND TESTICULAR LESIONS

### 4.1 Acute scrotal pain

**FIGURE 7:**
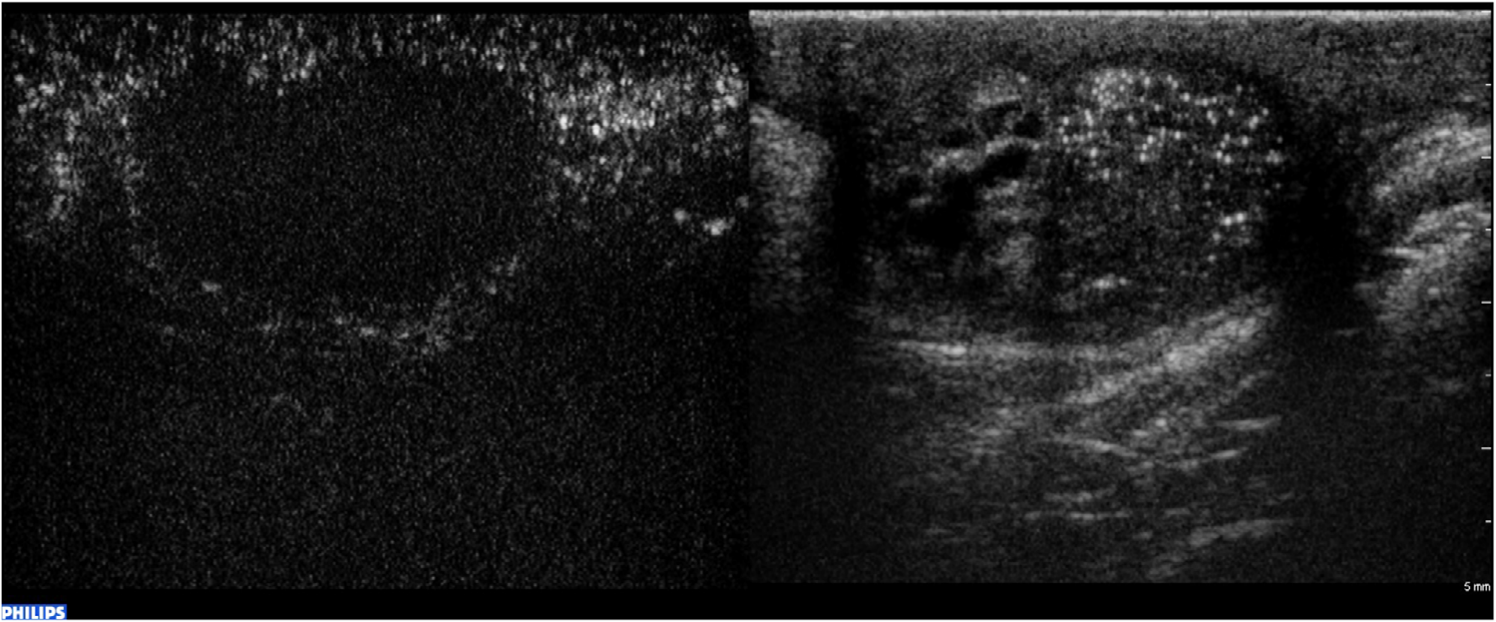
Testicular torsion. CEUS showed complete lack of enhancement of testis and spermatic cord in a patient with chronic (missed) torsion. Peri‐testicular tissues displayed increased vascularity on CEUS

**FIGURE 8:**
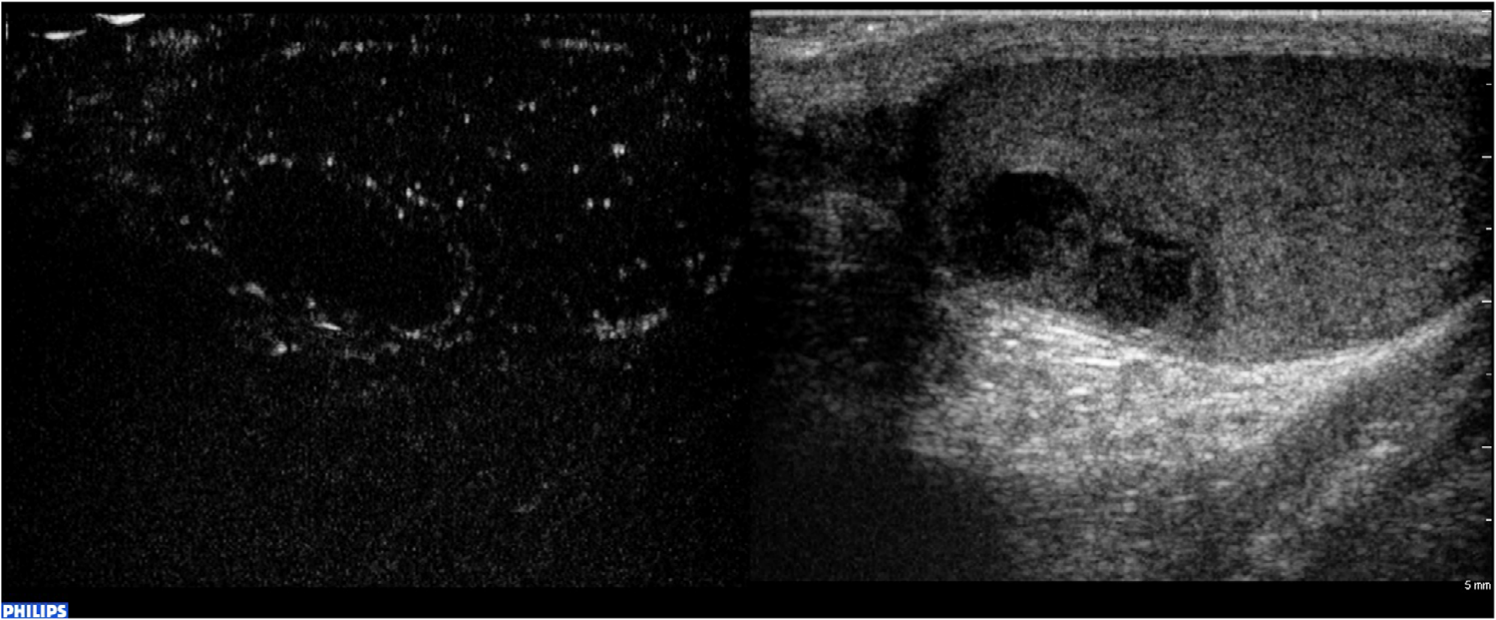
Intratesticular hematoma. CEUS appearances of intratesticular hematoma after blunt trauma. Dual‐display image showing contrast‐specific (left) and low MI B‐mode image (right). B‐mode image shows an intratesticular bilobated hypoechoic, avascular lesion. CEUS confirmed the absence of internal vascularity. Note the peri‐lesional hyperemia and the presence of internal echoes, representing artifact from echogenic content

Acute scrotal pain is a common urological emergency that requires a prompt diagnosis to determine the most appropriate treatment approach. Pain can be due to several causes, including epididymo‐orchitis, testicular torsion, testis’ appendix or epididymis torsion, intratesticular abscess, focal infarction, neoplasm, and trauma.^36^, ^68^ At first, diagnosis of clinical and medical history, associated with symptoms and biochemical assessment, is mandatory. Indeed, patients with testicular torsion usually present with symptoms of severe acute unilateral scrotal pain, nausea, and vomiting.^69^ US diagnostic images pertaining to testicular torsion are characterized by the absence of intratesticular blood flow at CD evaluation^70^ (Figure 7). In other cases, symptoms and clinical presentation might be similar among all causes of acute scrotal pain, whereas physical examination and laboratory evaluation may often not be exhaustive.^36^ Thus, CDUS could be helpful in investigating the underlying pain etiology.^68^, ^70^ Epididymitis and orchitis can be diagnosed by CDUS as a result of their typical clinical features. More specifically, epididymitis appears as an enlarged epididymis with distinct inflammatory signs such as increased vascularity CD and hydrocele.^70^ In contrast, orchitis is represented by an enlarged testis with decreased echogenicity and increased vascularity at CD.^70^ Other lesions such as abscess, hematoma, and infarction appear as hypoechoic lesions with absent vascularity (Figure 8),^70^ and their differential diagnosis should be done with testicular neoplasm, as previously reported.

Due to its ability to visualize microcirculation, CEUS can be helpful in various emergency situations. In 2009, Moschouris et al. published a preliminary research study that investigated the use of CEUS in patients with acute scrotum.^71^ On the basis of their 19 cases, authors concluded that CEUS had generally no advantages over CDUS and that this method could only be useful in patients with trauma. Recently, in a prospective study that included 50 patients with acute scrotum, CEUS was found to be more accurate in definitive diagnosis showing higher sensibility and specificity compared to conventional US.^36^ Conventional US provided a definitive tumor diagnosis in 34/50 patients, whereas CEUS provided the same diagnosis in 48/50 patients; the sensitivity was 76% for CDUS and 96% for CEUS, respectively, whereas the specificity was 45% for CDUS and 100% for CEUS, respectively.^36^

Among all causes of acute scrotal pain, CEUS seems to be particularly helpful in blunt scrotal trauma.^35^, ^71^, ^72^ Lobianco et al. examined 40 consecutive patients for blunt scrotal trauma with CDUS and CEUS. In 24 patients with positive findings (including interruption of the tunica albuginea, testicular fracture, lacerocontusion, total testicular ischemia, incomplete ischemia, hamartomatosis, arteriovenous malformation, and hematocele), CEUS demonstrated a greater sensitivity toward the detection of testicular lesions caused by blunt scrotal trauma. This was particularly true for small lesions.^35^ Also, CEUS could efficiently help depict fracture lines which could usually not be seen using grayscale US.^16^

**FIGURE 9:**
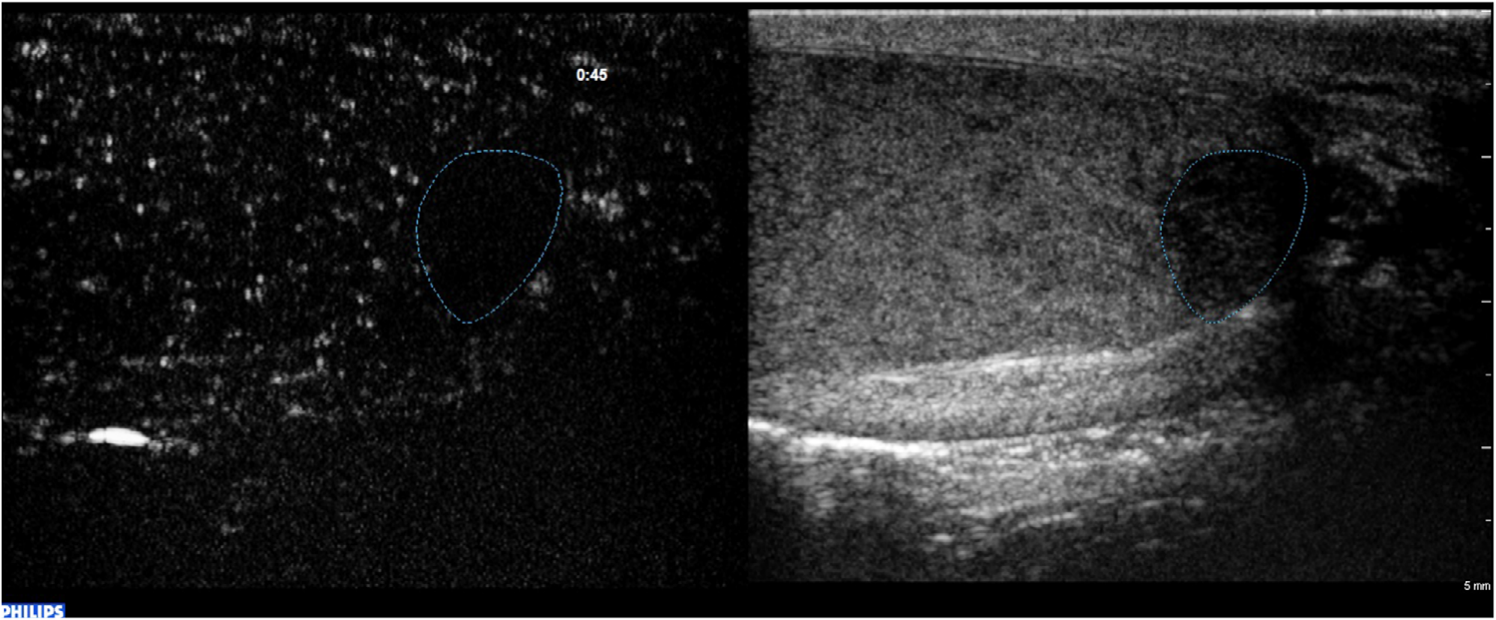
Focal ischemia. CEUS appearances of focal ischemia, confirmed at definitive histology. Dual‐display image showing contrast‐specific (left) and low MI B‐mode image (right). B‐mode image shows an intratesticular, well‐defined markedly hypoechoic, avascular lesion. CEUS confirmed the complete absence of internal vascularity and the patient, monorchid, underwent tissue sparing surgery

Another interesting field of application of CEUS in acute scrotal pain involves focal testicular infarction. In CDUS examination, testicular infarction typically appears as an avascular wedge‐shaped hypoechoic lesion.^73^ However, segmental testicular infarction can be round and resembling a TT,^74^ and sometimes presents a rim enhancement, probably due to granulation tissue in response to ischemic processes.^34^ In such cases, the patient's clinical history can help the clinician in the differential diagnosis process (Figure 9).

In a retrospective study, 20 men with acute scrotal pain, suspected of testicular infarction, were examined with CEUS. Compared with CDUS, CEUS facilitated improved lesion conspicuity, leading to the identification and recognition of ischemic lobules.^34^ Moreover, a perilesional rim enhancement was also identified by CEUS, which may represent a specific sign of subacute segmental testicular infarction.^34^, ^75^ The ability of CEUS in assessing the complete absence of vascularization with a rim enhancement has been used to distinguish a testicular hematoma^76^ or segmental testicular infarction^77^, ^78^, ^79^ from a tumor, in asymptomatic patients. Out of all the causes of scrotal pain, the use of CEUS has also been described in the diagnosis of spontaneous spermatic vein thrombosis, a rare condition which can lead to testicular pain and testicular swelling.^80^

### 4.2 Infertility

Nowadays, approximately 10% of infertile males are affected by nonobstructive azoospermia (NOA), which is characterized by a complete absence of spermatozoa in the seminal fluid due to minimal or no spermatogenesis.^81^ In some cases, the only therapeutic option in these patients is TESE. Unfortunately, sperm retrieval using conventional TESE has only proved to be successful in a subset of patients, approximately 50%, regardless of the cause of azoospermia (obstructive or nonobstructive).^39^, ^82^, ^83^, ^84^ Spermatogenesis is not equally distributed throughout the testis, and it would appear that sperm quality is better in areas with high tissue perfusion.^85^ The high accuracy of CEUS in assessing blood perfusion and microvascular architecture of the testes may suggest that this technique could help increase the probability of sperm retrieval.

To the best of our knowledge, a case report described for the first time that sperm quality and quantity depend on tissue perfusion within the testis.^86^ Since then, only two recent studies have focused on determining the usefulness of CEUS in infertility, by investigating whether it could be used to predict the success rate of testicular sperm retrieval techniques in infertile men.^37^, ^38^ Zhang et al. evaluated whether CEUS could be considered as a noninvasive approach for detecting the testicular area where spermatogenesis is most likely to be found in nonobstructive azoospermic testes. Among the 187 testes that underwent microdissection TESE, the sperm retrieval rates of the best perfusion area over the maximal longitudinal section were higher than those with the poorest perfusion area and conventional area where TESE is usually performed (63.1% vs. 34.7% and 47.1% respectively, *p* < 0.05). These findings suggest that spermatogenesis is not uniformly distributed throughout the testis because sperm quality is better in areas with high tissue perfusion. Moreover, the subsequent ROC analysis showed that W‐in ≤27 s, TTP ≤45 s, and PI ≥11 dB in the selected area could be considered the best cut‐off values for predicting positive sperm retrieval.^37^

In contrast, Xue et al. did not observe significant differences in the success rates of SR between the major and minor perfused areas in the 46 nonobstructive azoospermic patients examined. However, TESA had a very little chance of success in patients with NOA in case of: decreased intensity of the main perfusion area (defined as decreased intensity within 30 s after reaching the peak for both the main perfusion area and whole testis) with values < 8.6 dB; TTP of the whole testis  > 9.0 s; slope‐in of the whole testis  < 1.7 dB/s. Therefore, these quantitative CEUS features could have a negative predictive value on sperm retrieval.^38^

## 5 CONCLUSIONS

In conclusion, the literature underlines that CEUS is a safe, easy‐to‐perform, and cost‐effective diagnostic tool that is able to provide an accurate diagnosis in testicular lesions and in acute scrotal diseases when US findings are unclear. CEUS can increase diagnostic confidence levels, particularly in less experienced investigators. Therefore, CEUS should be proposed in every case where US diagnosis remains inconclusive, namely in the differential diagnosis of small testicular lesions to facilitate greater confidence in terms of selecting the appropriate patient intervention. Lesion enhancement indeed seems to have a high predictive value in the identification of neoplastic lesions. Similarly, the complete absence of enhancement can be interpreted as strong evidence for benignity, although some exceptions must be carefully considered. Literature on quantitative analysis is still scanty, particularly when distinguishing benign from malignant neoplasms. Further studies with larger cohorts are definitively required to refine the differential diagnosis between benign and malignant neoplasms. CEUS can also play an essential role in cases of acute scrotum, by excluding infarction and trauma, when testicular torsion cannot be defined. Finally, these interesting preliminary results can instigate the development of innovative research studies on pre‐TESE testicular perfusion to increase the chances of sperm recovery.

## CONFLICT OF INTEREST

The authors declare no conflict of interest regarding the publication of this article.

## AUTHOR CONTRIBUTION

All authors contributed to the conception and design of the review. Marta Tenuta, Franz Sesti, Ilaria Bonaventura, and Paola Mazzotta revised the literature and acquired the respective data. Marta Tenuta, Franz Sesti, and Ilaria Bonaventura wrote the first draft and designed this study's tables and figures. Carlotta Pozza and Marta Tenuta performed a first revision and synthesis of the manuscript. Riccardo Pofi and Paola Mazzotta were involved in a second critical revision of data. Carlotta Pozza and Daniele Gianfrilli performed the last critical revision for important intellectual content and granted the final approval of the version to be published. All authors are accountable for the accuracy and integrity of the work, and they all reviewed and approved the final manuscript.
